# Deep Learning-Based 3D Gravity Inversion with Well-Logging Prior Information

**DOI:** 10.3390/s26165012

**Published:** 2026-08-07

**Authors:** Mei-Ting Cai, Yu-Jie Zhang, Ao-Fei Jiang, Li He

**Affiliations:** School of Mathematics and Physics, China University of Geosciences (Wuhan), Wuhan 430074, China; caimeiting@cug.edu.cn (M.-T.C.); jiangaofei@cug.edu.cn (A.-F.J.); heli24@cug.edu.cn (L.H.)

**Keywords:** attention mechanism, deep learning, gravity inversion, well-logging constraints

## Abstract

As a fundamental technique in geophysical exploration, gravity inversion plays a crucial role in geological structure interpretation and mineral resource assessment. Recent advances in deep learning, particularly the remarkable capabilities of convolutional neural networks (CNNs) in image recognition, object detection, and semantic segmentation, have provided innovative solutions to nonlinear inverse problems in geophysics. However, conventional data-driven approaches often yield physically unrealistic inversion results due to the inherent non-uniqueness of solutions. This study proposes a novel 3D deep-learning gravity inversion framework incorporating well-logging prior constraints, which establishes a density-position relationship-based constraint mechanism through synergistic integration of surface gravity anomaly data and downhole petrophysical parameters. We develop a new neural network architecture that incorporates well-logging data as hard constraints for gravity inversion while preserving both density characteristics and spatial information of subsurface formations. Significantly, we implement the Convolutional Block Attention Module (CBAM) during network optimization, enabling selective enhancement of lithological features during backpropagation. This attention-guided mechanism achieves robust coupling between potential field anomalies and petrophysical parameters from well logs, substantially improving the spatial accuracy of 3D density reconstruction. Numerical experiments demonstrate that our method can accurately recover the density distribution of subsurface ore bodies. Comparative results show that the integration of well-logging information significantly enhances both solution reliability and structural consistency when compared to purely surface data-driven approaches. In practical application to field data from the San Nicolas sulfide deposit in Mexico, the proposed method outperforms conventional UNet-based approaches in terms of inversion performance.

## 1. Introduction

As one of the most fundamental geophysical exploration methods, gravity inversion plays an indispensable role in characterizing geological structures and assessing mineral resources [1,2]. Geophysical inversion techniques aim to reconstruct subsurface property distributions by establishing quantitative physical relationships between observed field data and underground attributes. Methodologically, these techniques can be classified into two principal categories: linear and nonlinear inversion paradigms [3]. While both approaches and their associated optimization algorithms have been widely adopted in conventional gravity inversion practices [4,5,6,7], they face two significant challenges when applied to 3D gravity inversion: prohibitively high computational costs, and degraded spatial resolution in the resulting models, particularly evident in their inability to accurately delineate geological boundaries [8].

The rapid development of neural networks, particularly convolutional neural networks (CNNs), has revolutionized their application in geophysics, both for enhancing inversion results and solving geophysical problems directly [9,10,11,12]. The fundamental objective of gravity inversion is to estimate 3D subsurface density distributions from 2D surface gravity anomaly measurements—a process conceptually similar to 2D-to-3D image reconstruction in computer vision. Recent years have witnessed growing interest in applying deep learning approaches to advance 3D gravity inversion methodologies [13,14,15]. Yang et al. [16] pioneered the use of UNet architectures to establish direct mappings from 2D gravity data to 3D model space through depth-stratified representations. Zhang et al. [17] developed an enhanced UNet architecture incorporating residual connections and channel attention mechanisms for gravity gradient inversion using variable-geometry prismatic models. Another significant contribution by Zhang et al. [18] introduced an innovative dimensionality transformation network combining an encoder for 3D latent space projection with a physics-constrained decoder for complete 3D geological reconstruction. Huang et al. [19] employed stochastic “Random Walk” techniques to generate comprehensive training datasets, systematically evaluating UNet generalization capabilities under complex geological conditions. Most recently, Lv et al. [20] proposed a UNet3+-based multitask framework that integrates physical constraints with uncertainty quantification to overcome the limited generalizability of conventional methods while enabling rapid forward modeling and inversion.

Although data-driven inversion methods have achieved notable success, inversions based solely on gravity anomaly data remain inherently non-unique, which may lead to ambiguous or geologically inconsistent subsurface models [21,22]. Incorporating complementary geophysical observations and structural constraints is therefore an effective means of reducing inversion ambiguity and improving the structural consistency and reliability of the recovered models [23]. In this context, geological and geophysical prior information can be introduced to further constrain the inversion process. In particular, well-logging data provide direct, depth-resolved measurements of subsurface geological and petrophysical properties and can therefore serve as valuable prior constraints for gravity inversion [24,25,26,27]. Recent studies have attempted to combine well-logging information with deep learning methods to enhance inversion accuracy and reliability. Li et al. [28] developed a self-supervised network framework incorporating well-logging constraints; however, the 1D integration format failed to fully exploit spatial distribution characteristics, limiting 3D geological structure characterization. This research trend has also emerged in recent studies on seismic inversion and multimodal geophysical inversion, where well-logging information, geological priors, and physical constraints have been incorporated to improve the reliability and geological consistency of inversion results [29,30,31]. To overcome the limitation of 1D well-logging information integration in 3D gravity inversion, our study spatially maps well-logging prior information into 3D space while preserving positional attributes before constrained inversion. The mapped 3D data are then jointly input with surface gravity anomalies during network training. This approach transforms the originally isolated 1D well-logging information into a spatially distributed prior constraint, thereby more effectively constraining the inversion mapping from 2D gravity anomalies to 3D density structures, reducing the interpretation ambiguity caused by the non-uniqueness of gravity inversion, and ultimately enhancing the reliability and accuracy of interpretation results.

The main contributions of this study are summarized as follows. First, the one-dimensional well-logging density profile is spatially mapped into the three-dimensional model domain according to the borehole location and depth, thereby preserving the density–depth–position relationship and transforming the isolated vertical profile into a spatially explicit prior for three-dimensional gravity inversion. Second, an attention-guided feature-fusion mechanism is incorporated into the encoder to progressively integrate the mapped well-logging prior with multiscale gravity-anomaly features. The channel- and spatial-attention branches adaptively emphasize feature channels and spatial regions associated with the borehole information and anomalous density structures, rather than relying solely on direct input concatenation. Third, a jointly constrained inversion framework is established by using the spatially mapped well-logging information as an explicit prior, the Dice loss as a structural constraint and the gravity-forward misfit as a physical-consistency constraint. This coordinated design helps alleviate the ambiguity of gravity-only inversion and enhances the vertical resolution and structural reliability of the reconstructed density models.

The remainder of this paper is organized as follows: Section 2 comprehensively describes the proposed inversion methodology, including model architecture, loss function formulation, and optimization strategy. Section 3 presents experimental results from both synthetic and field datasets, accompanied by comparative analyses with conventional gravity inversion approaches. Conclusions are detailed in Section 4.

## 2. Methods

### 2.1. Theoretical Framework

**Figure 1:**
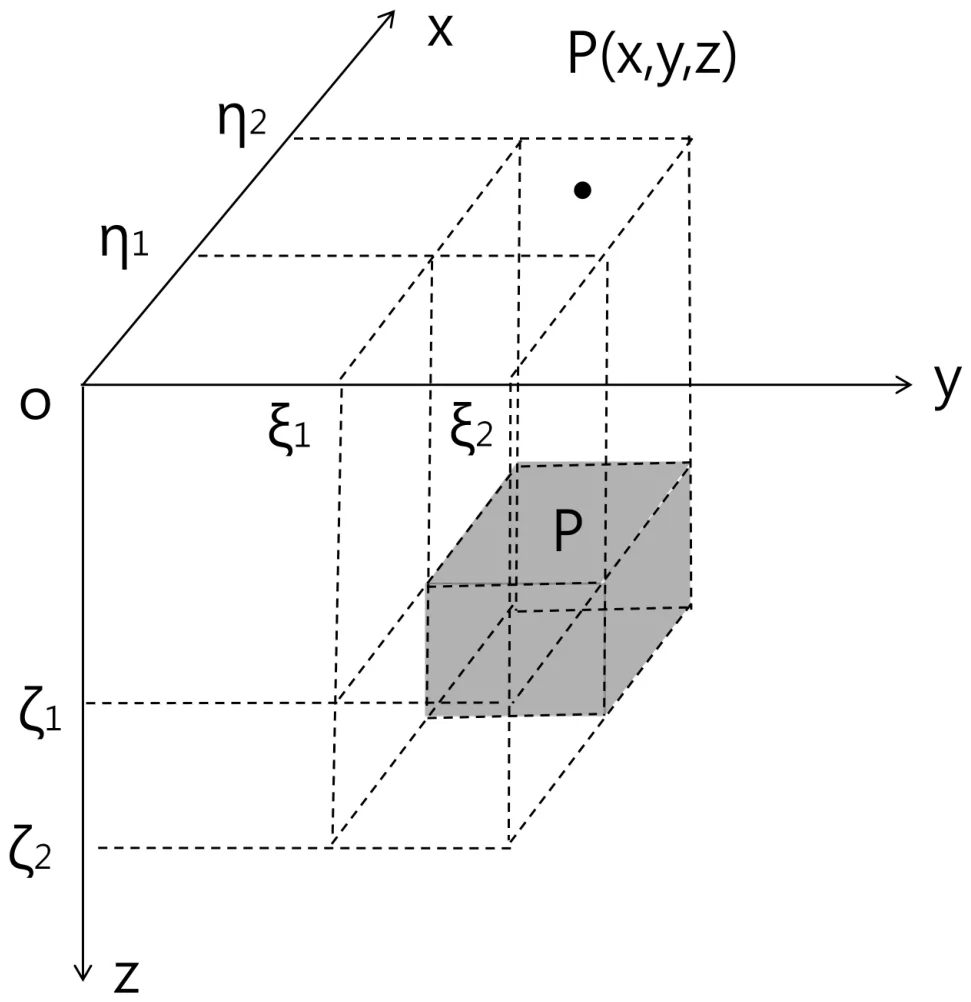
Schematic diagram of gravity anomaly computation. The gray prism denotes a representative discretized subsurface cell.

Gravity forward modeling calculates surface gravity anomalies through fundamental physical equations [32]. In practical implementations, subsurface anomalous bodies are conventionally discretized into multiple rectangular prismatic cells as illustrated in Figure 1. The gravitational contribution from each individual cell to observation points is computed, with the total anomaly field obtained through superposition of all cellular contributions. When the discretization is sufficiently fine (i.e., with adequate cell numbers and sufficiently small volumes), this approach can effectively approximate geological bodies of arbitrary geometry. Consequently, the relationship between surface gravity anomalies and subsurface density distributions can be mathematically expressed as [33]:

(1) d=Gm,

where d denotes an x×1 vector comprising gravity anomaly values at all observation points, expressed as $d={\left(d_{1},d_{2},\ldots ,d_{x}\right)}^{T}$; m represents a vector containing the density values of the discretized geological units, formulated as $m={\left(m_{1},m_{2},\ldots ,m_{y}\right)}^{T}$. Here, the density values are expressed relative to the reference background density. Since the reference background density is taken as the zero level in the numerical models, $m_{j}$ is numerically equivalent to the density contrast of the *j*-th cell. The sensitivity matrix $G={\left[g_{ij}\right]}_{x\times y}$, with dimensions x×y, consists of sensitivity coefficients, where $g_{ij}$ represents the gravitational response at the *i*-th surface observation point generated by the *j*-th subsurface rectangular prism with a unit density contrast.

Data-driven 3D gravity inversion represents a methodology that inintegrates deep learning techniques with inversion theory to directly infer subsurface density models from observed gravity data. This process involves three critical phases:

(i) Generation of sufficient subsurface density models as training labels.
(ii) Computation of theoretical gravity anomalies through forward modeling using (1).
(iii) Training of neural networks to establish complex nonlinear mappings between gravity anomalies and density models, thereby achieving high-fidelity reconstruction of subsurface structures.

The mapping relationship can be mathematically formulated as:

(2) m=F(d;θ),

where m corresponds to the 3D subsurface density model, d denotes the input 2D gravity anomaly data, and θ signifies the trainable parameters of the neural network. Through optimization algorithms, θ is iteratively adjusted to ensure the mapping function *F* accurately characterizes subsurface structural features.

To enhance the network’s generalization capability and ensure physically plausible inversion results with improved practical relevance, we reformulate the mapping relationship in (2) by incorporating well-logging prior constraints:

(3) m=F(d,L;θ),

where L denotes the spatially mapped well-logging prior information. Let $\ell ={\left(\ell_{1},\ell_{2},\ldots ,\ell_{N_{z}}\right)}^{T}$ denote the original one-dimensional well-logging density profile, where $\ell_{k}$ is the density value corresponding to the *k*-th depth layer. The horizontal coordinates of the borehole are converted into the corresponding grid indices $\left(i_{w},j_{w}\right)$ according to the model discretization. The one-dimensional well-logging profile is then embedded into the three-dimensional model grid as

(4) $$L_{k,i,j}=\left\{\begin{array}{cc} \ell_{k}, & i=i_{w},\;j=j_{w},\\ 0, & \mathrm{otherwise}, \end{array}\right.\;k=1,2,\ldots ,N_{z},$$

where $L\in R^{N_{z}\times N_{x}\times N_{y}}$ represents the mapped well-logging tensor. In this study, $N_{z}=16$ and $N_{x}=N_{y}=32$; therefore, L has dimensions of 16×32×32. This operation embeds the density value at each logging depth into the corresponding depth layer and horizontal borehole location, thereby preserving the density–depth–position relationship of the well-logging data. The resulting tensor is a sparse three-dimensional prior in which the well-logging values form a vertical density column at the borehole location.

The two-dimensional surface gravity anomaly is reshaped as a single-channel tensor $D\in R^{1\times N_{x}\times N_{y}}$. The gravity-anomaly tensor and the spatially mapped well-logging tensor are concatenated along the channel dimension:

(5) $$X={\mathrm{Concat}}_{c}\left(D,L\right),\;X\in R^{\left(N_{z}+1\right)\times N_{x}\times N_{y}},$$

where ${\mathrm{Concat}}_{c}\left(\cdot \right)$ denotes channel-wise concatenation. Accordingly, the input tensor of the proposed network has dimensions of 17×32×32. The first channel contains the surface gravity anomaly, whereas the remaining 16 channels represent the mapped well-logging information at the corresponding depth layers.

**Figure 2:**
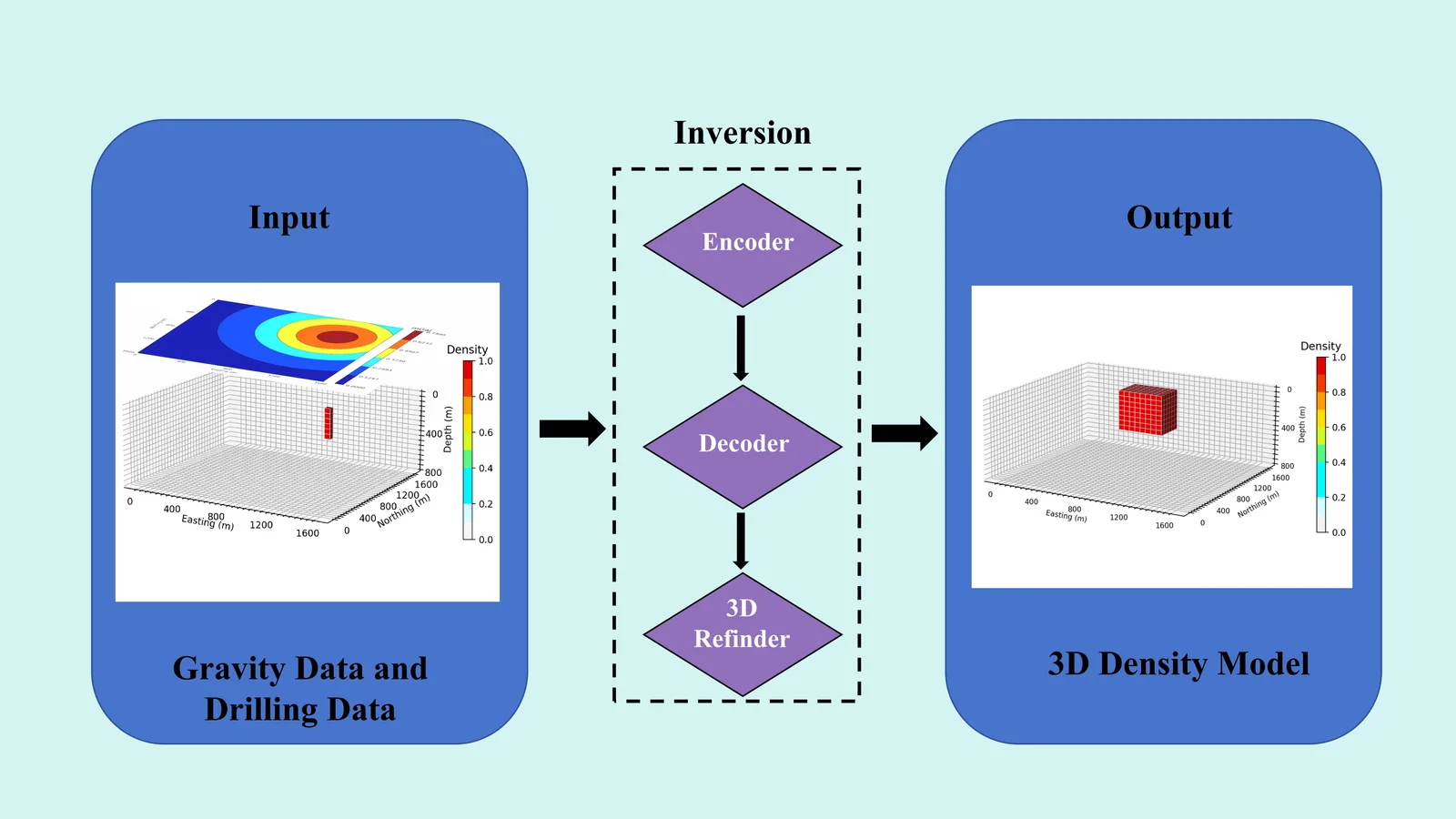
Gravity inversion workflow.

Based on the above spatial mapping and joint-input construction, the overall gravity inversion workflow implemented in this study is illustrated in Figure 2.

**Figure 3:**
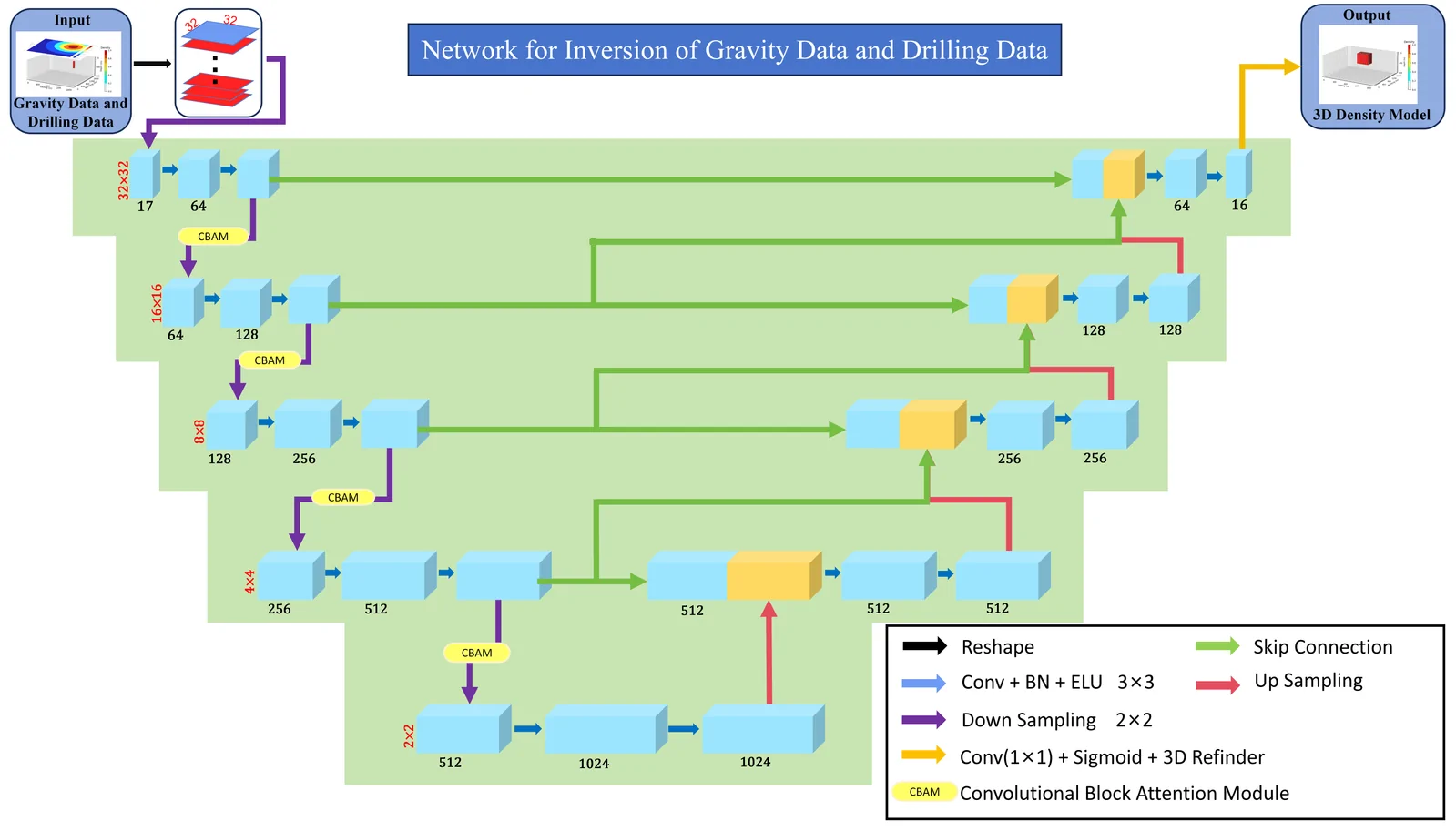
UCA-W network architecture for 3D gravity inversion.

**Figure 4:**
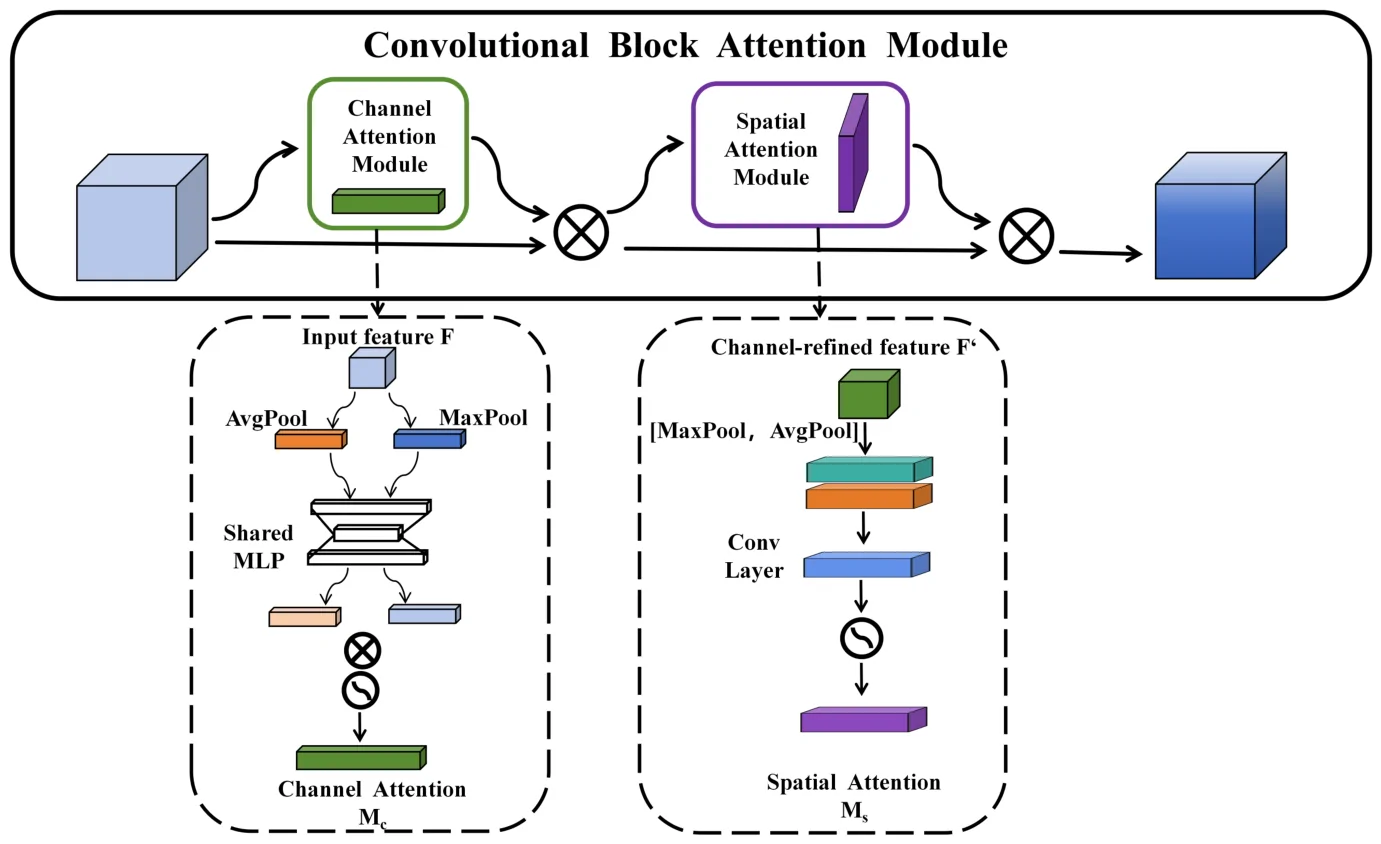
Convolutional Block Attention Module. Solid and dashed arrows denote feature propagation and links to detailed module structures, respectively; green and purple indicate channel and spatial attention, while other colors distinguish intermediate features and operations.

In computer vision applications, the U-shaped encoder-decoder architecture with skip connections (UNet) has demonstrated exceptional performance in image segmentation tasks by simultaneously achieving feature extraction and detail preservation [34]. Adopting a similar architectural framework, we developed an optimized gravity inversion network termed UCA-W (UNet with CBAM Attention and Well Constraints), as depicted in Figure 3. As defined in Equations (4) and (5), the encoder receives a 17×32×32 joint input tensor composed of the surface gravity anomaly and the spatially mapped well-logging prior. The depth dimension of the mapped well-logging tensor is treated as the channel dimension of the encoder, allowing the network to jointly extract regional gravity-field features and localized, depth-resolved density information. During downsampling operations, the network incorporates a Convolutional Block Attention Module (CBAM). As detailed in [35] and illustrated in Figure 4, CBAM implements dual attention pathways: channel attention and spatial attention modules. This architecture facilitates effective fusion between well-logging information and latent features extracted from gravity anomaly data, while imposing depth-wise constraints during feature integration.

During network training, the Dice loss function was employed to quantify the discrepancy between the theoretical density model m and the predicted model $\tilde{m}$. In the 3D density models considered in this study, anomalous-density cells occupy only a small proportion of the entire model, whereas most cells belong to the zero-valued background region. Under such an imbalanced spatial distribution, the contribution of the anomalous regions may be overwhelmed by the large number of background cells during model optimization. Moreover, the objective of gravity inversion is not only to recover density values but also to accurately delineate the locations, spatial extents, geometries, and boundaries of anomalous bodies. The Dice loss is therefore suited to this task because it directly measures the spatial overlap between the predicted and theoretical anomalous regions, reduces the dominance of background cells, and enhances the reconstruction of anomalous-body geometries and boundaries. While the Mean Squared Error (MSE) loss function was utilized to evaluate the misfit between the observed surface gravity anomalies d and the calculated anomalies $\tilde{d}$ derived from forward modeling of the predicted model.

(6) $$\mathrm{DiceLoss}=1-\frac{2\sum_{i=1}^{y}m_{i}{\tilde{m}}_{i}}{\sum_{i=1}^{y}m_{i}^{2}+\sum_{i=1}^{y}{\tilde{m}}_{i}^{2}},$$

(7) $$\mathrm{MSELoss}=\frac{1}{x}\sum_{i=1}^{x}{\left({\tilde{d}}_{i}-d_{i}\right)}^{2}.$$

The composite loss function of the network was formulated as:

(8) TotalLoss=αDiceLoss+βMSELoss,

where α and β denote the weighting coefficients of the Dice loss and MSE loss, respectively. Based on preliminary validation experiments using different coefficient combinations, the weights were empirically set to α=0.8 and β=0.2. A larger weight was assigned to the Dice loss because the recovery of sparse anomalous-body geometries is particularly susceptible to the dominance of background cells. Meanwhile, the MSE term was retained to constrain the discrepancy between the observed gravity anomalies and those calculated through forward modeling of the predicted density model. This weighting combination provides a reasonable balance between structural reconstruction and gravity-data consistency.

### 2.2. Evaluation Metrics

In three-dimensional gravity inversion, subsurface density models can be divided into two components: the background density model and the source density model. The majority of subsurface components belong to the background density model. To evaluate the spatial distribution of both background and source densities, the Average Accuracy (ACC) metric is employed. Additionally, the Mean Absolute Error (MAE), Intersection Over Union (IOU), and Structural Similarity Index Measure (SSIM) are utilized as supplementary evaluation metrics.

(9) $$\mathrm{ACC}=\frac{C(|{\tilde{m}}_{i}-m_{i}|<t)}{y},$$

(10) $$\mathrm{MAE}=\frac{1}{y}\sum_{i=1}^{y}\left|{\tilde{m}}_{i}-m_{i}\right|,$$

(11) $$\mathrm{IOU}=\frac{\sum_{i=1}^{y}\left|{\tilde{m}}_{i}\cap m_{i}\right|}{\sum_{i=1}^{y}\left|{\tilde{m}}_{i}\cup m_{i}\right|},$$

(12) $$\mathrm{SSIM}=\frac{\left(2\mu_{m}\mu_{\tilde{m}}+C_{1}\right)\left(2\sigma_{m\tilde{m}}+C_{2}\right)}{\left(\mu_{m}^{2}+\mu_{\tilde{m}}^{2}+C_{1}\right)\left(\sigma_{m}^{2}+\sigma_{\tilde{m}}^{2}+C_{2}\right)},$$

where m and $\tilde{m}$ represent the theoretical model vector and predicted model vector, respectively; ${\tilde{m}}_{i}$ and $m_{i}$ denote the *i*-th elements of the predicted and theoretical model vectors; *y* indicates the dimensionality of vector m; and *t* is the predefined threshold. When the absolute error between ${\tilde{m}}_{i}$ and $m_{i}$ satisfies $|{\tilde{m}}_{i}-m_{i}|<t$, the prediction is considered accurate. *C* is used to count the number of correctly predicted elements. The accuracy value is obtained by dividing the number of correctly predicted elements by the discretization level of the total grid for the anomalous body. In this study, the threshold value is set to 0.01. The MAE reflects the average error between the reconstructed model and the theoretical model. The IOU indicates the overlap between the predicted subsurface boundaries and the true subsurface boundaries. A higher IOU value demonstrates better alignment between predicted and actual regions, reflecting improved model accuracy. The SSIM is defined with the following components: $\mu_{m}$ and $\mu_{\tilde{m}}$ represent the mean values of m and $\tilde{m}$, respectively; $\sigma_{m}$ and $\sigma_{\tilde{m}}$ denote the standard deviations of m and $\tilde{m}$; $\sigma_{m\tilde{m}}$ is the covariance between m and $\tilde{m}$; and $C_{1}$ and $C_{2}$ are predefined constants. The SSIM value ranges from [−1,1], effectively evaluating the similarity between the reconstructed subsurface model and the true subsurface model, particularly in capturing geological structural details and edge features.

### 2.3. Construction of the Training Dataset

The optimal methodology for constructing training datasets relies on utilizing extensive real-world data samples. However, acquiring paired field data comprising surface gravity anomalies, three-dimensional subsurface density distributions, and well-logging information presents significant practical challenges. In this investigation, the subsurface domain of the study area was discretized into 16,384 cubic cells (16 × 32 × 32), with each cell having an edge length of 50 m. Gravity observation data were simulated on a 32 × 32 surface grid with a spacing of 50 m, and the density contrast of the anomalous sources was uniformly set to 1 g/cm^3^.

Following the stochastic random-walk synthesis approach described by Huang et al. [19], the subsurface density models were generated from one or two randomly initialized seed bodies. Each seed body initially consisted of 2 × 2 × 2 adjacent cells and was subsequently translated through the model domain. At each walking step, one of the three Cartesian axes and one of the two corresponding movement directions were randomly selected, resulting in six possible propagation directions. The seed body was shifted by two grid cells at each step and constrained to remain within the prescribed horizontal and vertical model boundaries. For single-source models, the total number of walking steps was randomly selected between 60 and 80. For dual-source models, two independent random walks were generated, with 30–40 steps assigned to each source. Variations in the initial positions, propagation directions, and walking trajectories produced spatially connected anomalous bodies with different horizontal locations, burial depths, spatial extents, orientations, and irregular boundaries. Compared with datasets composed exclusively of regular geometric bodies, this procedure provides greater structural diversity and reduces the model’s dependence on predefined regular geometries.

**Figure 5:**
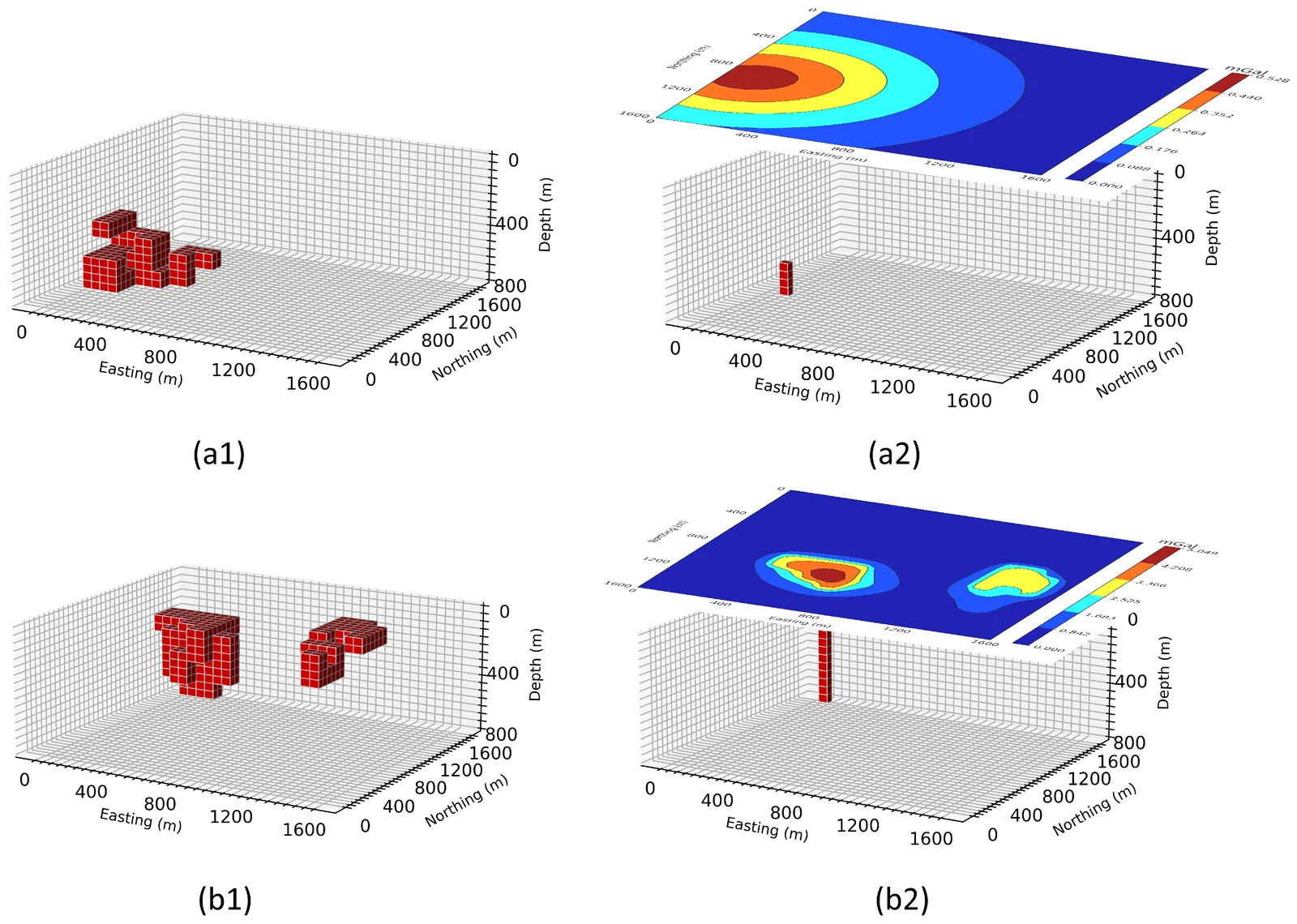
Density model samples from the training dataset. (**a1**) Single-source density model generated from one random-walk seed; (**a2**) corresponding gravity anomaly and well-logging data for the single-source density model; (**b1**) dual-source density model generated from two independent random-walk seeds; (**b2**) corresponding gravity anomaly and well-logging data for the dual-source density model.

For each generated density model, the corresponding theoretical gravity anomalies were calculated using (1), thereby ensuring that the input gravity data strictly satisfied the governing physical relationship between subsurface density distributions and surface gravity responses. The horizontal coordinates associated with the maximum gravity anomaly were designated as representative well-logging locations, and the corresponding vertical density profiles were directly extracted from the same three-dimensional density model as prior constraints. Consequently, the gravity anomalies, well-logging information, and target density models in each sample were physically consistent and spatially registered. Figure 5 presents representative single-source and dual-source models from the training dataset.

A total of 22,600 subsurface density models were generated in this study, encompassing both single-source and dual-source geological configurations to comprehensively represent geological structures with diverse geometrical characteristics. The corresponding theoretical gravity anomalies and well-logging data were computed following the aforementioned methodology. The dataset was systematically partitioned into 20,000 training samples, 2000 testing samples, and 600 validation samples to ensure robust model evaluation.

## 3. Examples

### 3.1. Synthetic Example

To validate the effectiveness of well-logging constrained gravity inversion and demonstrate the superiority of the proposed UCA-W network, three synthetic experiments were conducted: (1) comparative analysis, (2) well-position offset test, and (3) multi-density body scenario.

The computations were performed on a workstation equipped with a 6-core Intel Xeon w3-2425 CPU (Intel Corporation, Santa Clara, CA, USA), 128 GB RAM, and two NVIDIA GeForce RTX 4090 GPUs (NVIDIA Corporation, Santa Clara, CA, USA) (24 GB VRAM each). The network was implemented using Python (v3.9.18) and PyTorch (v2.4.1+cu121, CUDA 12.1). During network training, hyperparameters were set as follows: 200 epochs, batch size of 32, learning rate of $3\times 10^{-4}$, with the Adam optimizer employed for parameter updating. An early stopping mechanism was implemented to enhance model performance, optimize training efficiency, and mitigate overfitting risks.

**Figure 6:**
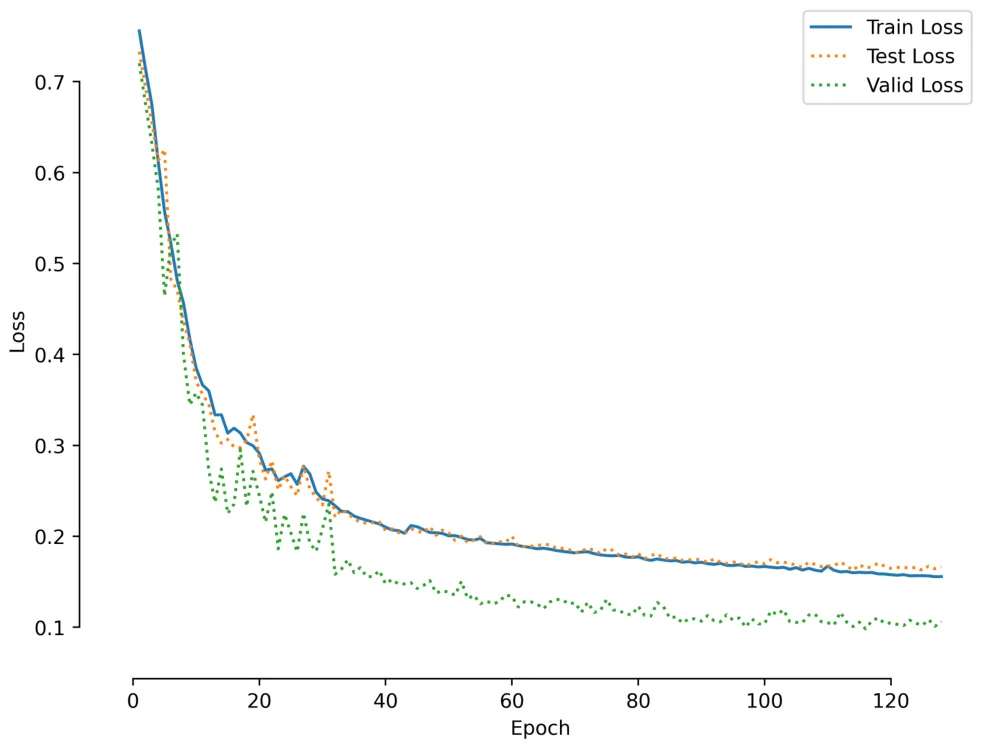
Training, testing, and validation loss curves of the proposed network.

Figure 6 illustrates the loss function curves of the UCA-W network, where the consistent convergence trends across training, validation, and test sets confirm the model’s robust generalization capability without overfitting.

#### 3.1.1. Example 1: Network Architecture Comparison

In this numerical experiment, we conducted comprehensive model training on the constructed synthetic dataset. Following the evaluation metrics detailed in Section 2.2, Table 1 systematically compares the performance of four distinct network architectures on the simulated training dataset. The compared models include: UNet-W (UNet incorporating well-logging constraints), SSGI-W (the method proposed by Li et al. [28]), and UCA-W (our proposed network). To rigorously evaluate the contribution of well-logging information, we additionally tested the UCA network without well-logging constraints. The quantitative results demonstrate that the UCA-W network with well-logging prior constraints achieves superior inversion accuracy, particularly in terms of ACC and SSIM metrics. These findings not only validate the effectiveness of our proposed method but also quantitatively confirm the critical role of well-logging data in enhancing subsurface model reconstruction.

**Figure 7:**
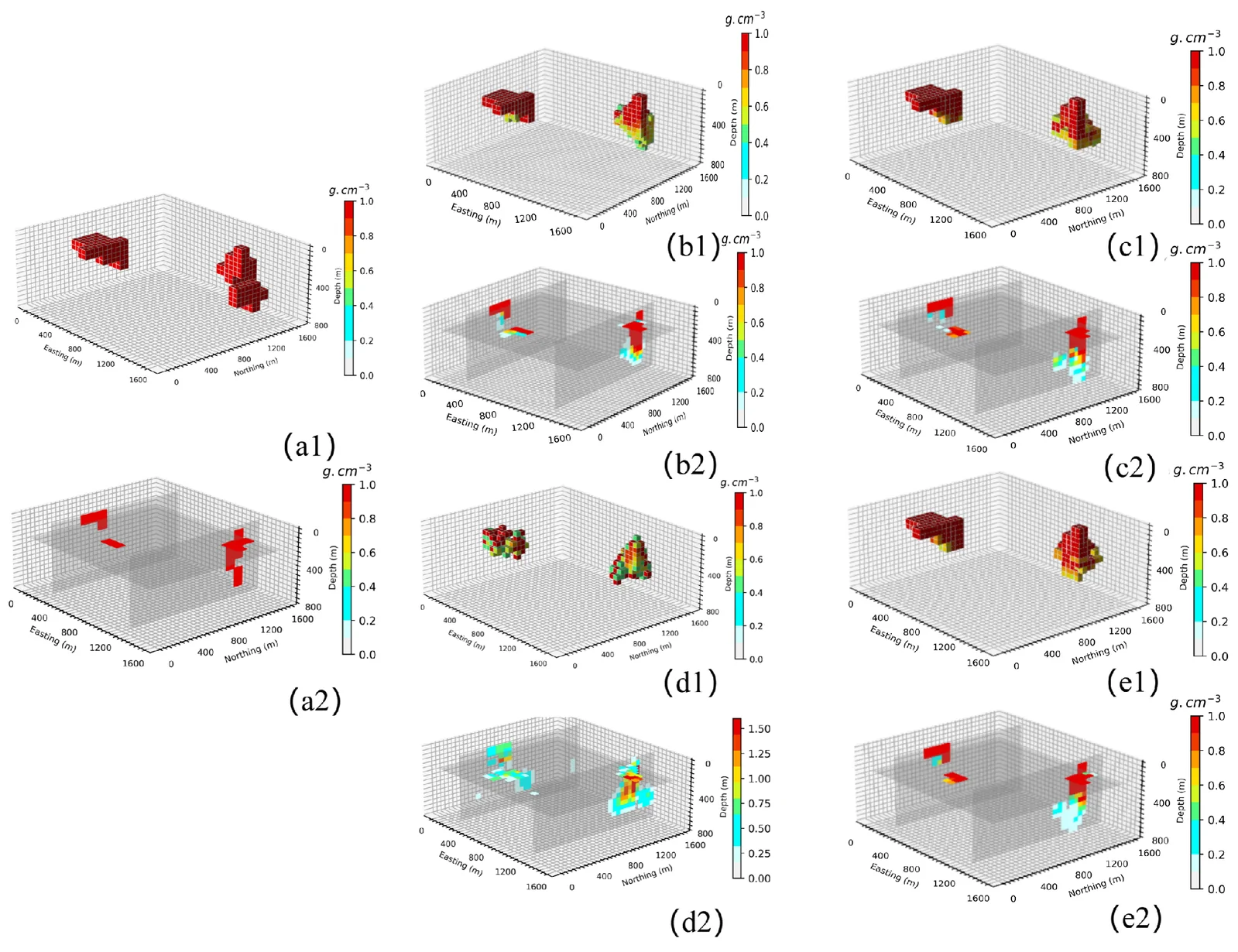
Comparison of inversion results for a random model with two density bodies using four different networks: (**a1**) actual subsurface density distribution; (**a2**) cross-sectional views at Depth = 300 m and Easting = 300 m, 1300 m; (**b1**) subsurface density distribution predicted by the UCA-W network; (**b2**) cross-sectional views at Depth = 300 m and Easting = 300 m, 1300 m predicted by the UCA-W network; (**c1**) subsurface density distribution predicted by the UNet-W network; (**c2**) cross-sectional views at Depth = 300 m and Easting = 300 m, 1300 m predicted by the UNet-W network; (**d1**) subsurface density distribution predicted by the SSGI-W network; (**d2**) cross-sectional views at Depth = 300 m and Easting = 300 m, 1300 m predicted by the SSGI-W network; (**e1**) subsurface density distribution predicted by the UCA network; (**e2**) cross-sectional views at Depth = 300 m and Easting = 300 m, 1300 m predicted by the UCA network.

Figure 7 presents the inversion results and corresponding cross-sections for a random model containing two density bodies, obtained using the four networks. The left density body represents a shallow structure, while the right one corresponds to a deeper formation. Notably, the SSGI-W network exhibits significant inversion failures, which may be attributed to the limited adaptability of Li et al.’s [28] method to our dataset. For the shallow density body (left), all three networks (UCA-W, UNet-W, and UCA) successfully recovered both the density values and boundary information. However, for the deeper density body (right), the UCA-W network outperforms both UNet-W and UCA networks in accurately delineating the density structure along the depth direction, with significantly reduced artifacts. This observation suggests that the integration of well-logging constraints effectively improves the imaging quality of deep geological structures.

#### 3.1.2. Example 2: Well-Logging Position Offset Test

**Figure 8:**
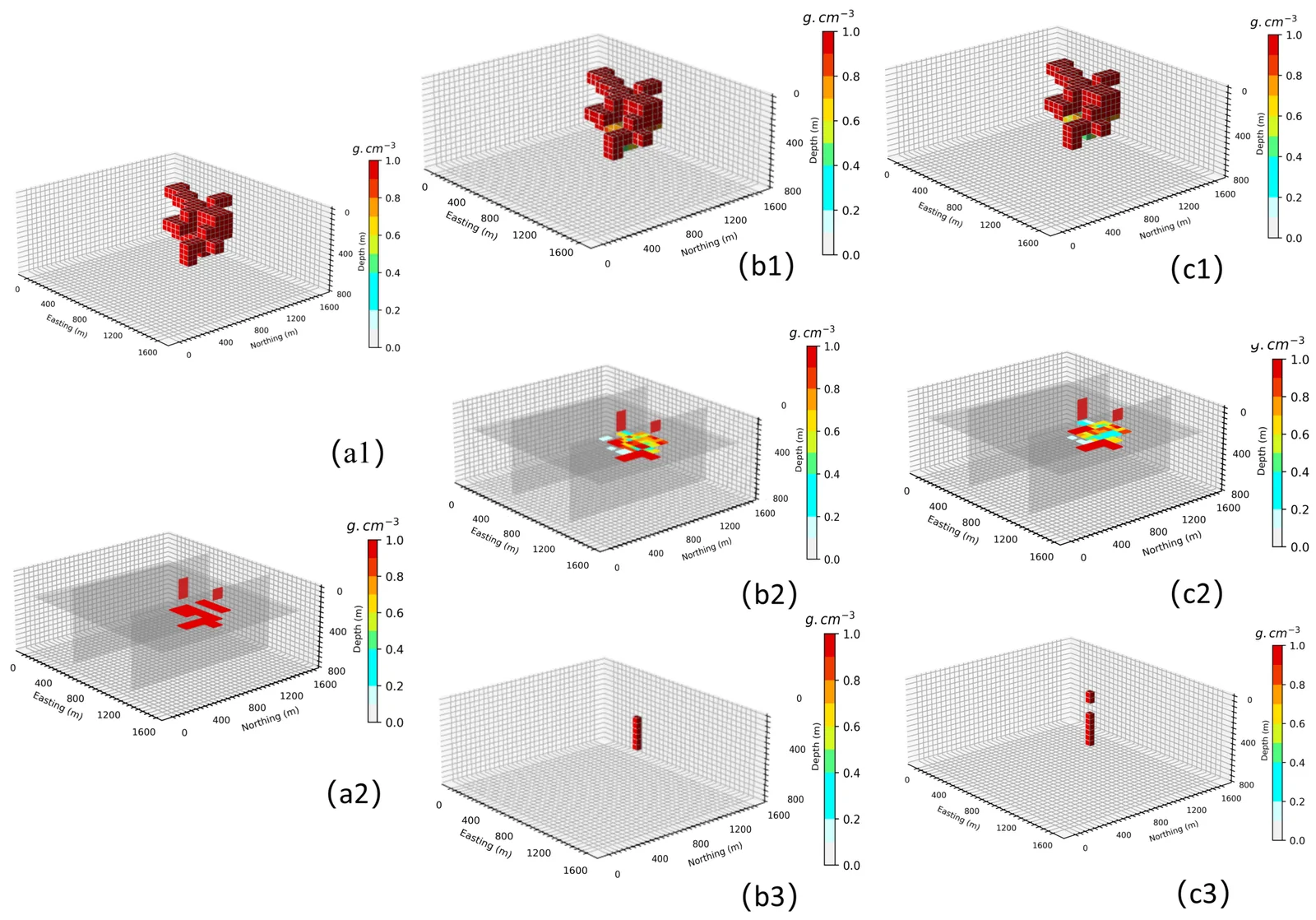
Comparison of inversion results for two density bodies before and after offsetting the well-logging position: (**a1**) actual subsurface density distribution; (**a2**) cross-sectional views at Depth = 300 m and Easting = 300 m, 1300 m for the actual distribution; (**b1**) predicted subsurface density distribution without offsetting the well-logging position; (**b2**) cross-sectional views at Depth = 300 m and Easting = 300 m, 1300 m for the prediction without offsetting; (**b3**) well-logging information without offsetting; (**c1**) predicted subsurface density distribution after offsetting the well-logging position; (**c2**) cross-sectional views at Depth = 300 m and Easting = 300 m, 1300 m for the prediction after offsetting; (**c3**) well-logging information after offsetting.

In practical mining applications, the optimal well-logging position may deviate from the surface gravity anomaly extremum due to various factors including topographic complexity, environmental constraints, and operational considerations. To evaluate our network’s robustness to such deviations, we designed a simulation experiment by introducing random offsets (three units in both horizontal and vertical directions) to the well-logging position. The inversion results for two density bodies under this condition are displayed in Figure 8.

The experimental results confirm that our method maintains satisfactory inversion performance even with positional deviations of well-logging data. This finding holds significant practical implications, demonstrating the method’s adaptability to common scenarios in field exploration. While positional offsets introduce certain inversion errors, the overall spatial distribution characteristics of the density structures remain well reconstructed. This experiment successfully verifies the robustness of our approach in handling real-world complexities.

#### 3.1.3. Example 3: Multiple Density Bodies Validation

**Figure 9:**
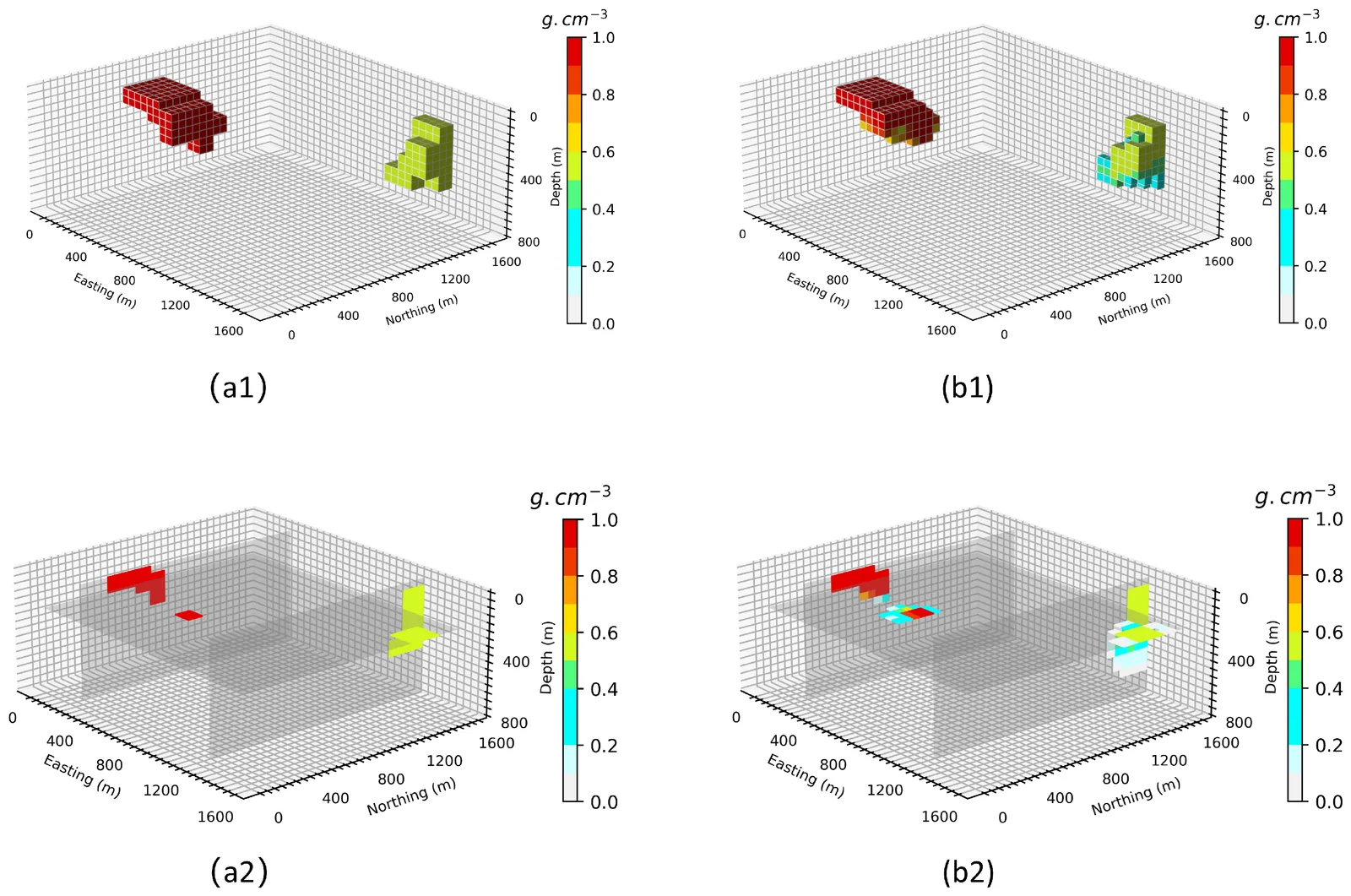
Inversion results of a random model with two different density bodies: (**a1**) actual subsurface density distribution; (**b1**) subsurface density distribution predicted by the UCA-W network; (**a2**) cross-sectional views at Depth = 300 m and Easting = 300 m, 1300 m; (**b2**) cross-sectional views at Depth = 300 m and Easting = 300 m, 1300 m predicted by the UCA-W network.

To further assess the method’s capability in handling complex geological structures, we constructed a synthetic model containing two density bodies with contrasts of 1 g/cm^3^ and 0.6 g/cm^3^, respectively, simulating typical lithological interfaces in real geological settings. The corresponding inversion results are presented in Figure 9.

The results demonstrate our method’s excellent adaptability in multi-density-body inversion, maintaining high prediction accuracy through three key aspects: (1) clear differentiation between density boundaries, (2) accurate reconstruction of geometric shapes, and (3) reliable recovery of target density values. Such capabilities are particularly valuable for mineral resource assessment in complex geological conditions. The experiment confirms that our deep learning framework incorporating well-logging constraints can effectively handle geological scenarios with multiple density bodies, providing reliable technical support for practical mineral exploration.

#### 3.1.4. Example 4: Noise Robustness Experiment

**Figure 10:**
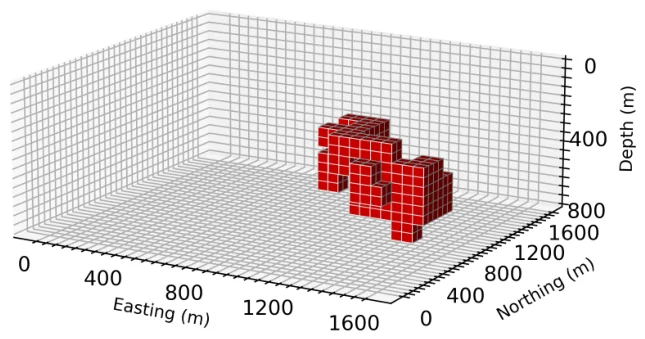
True density model.

**Figure 11:**
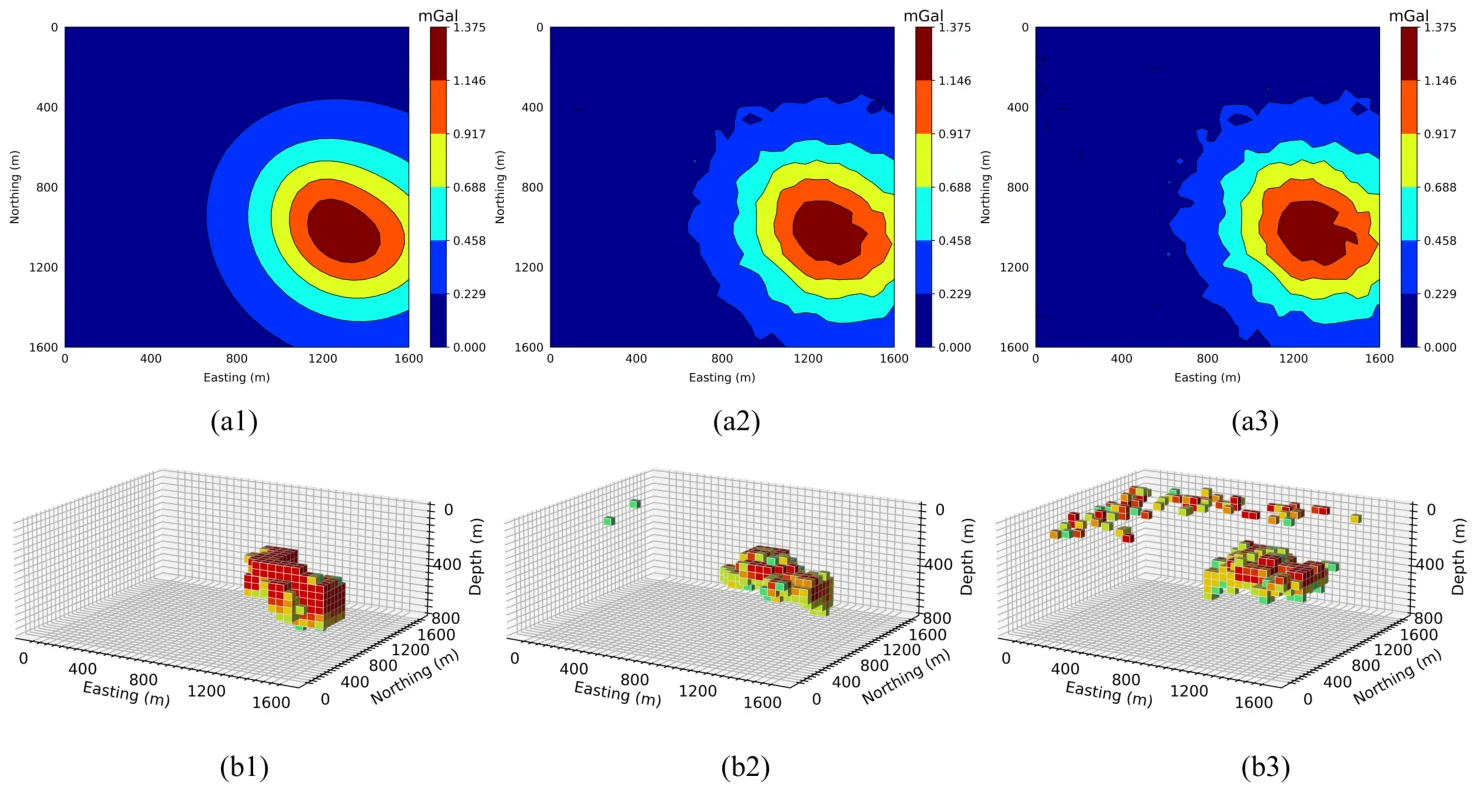
Gravity anomalies and corresponding inversion results under different Gaussian white-noise levels. (**a1**–**a3**) Gravity anomaly maps under noise levels of 0%, 2%, and 3%, respectively; (**b1**–**b3**) corresponding inversion results. Only voxels with predicted values greater than 0.5 are displayed. The well-logging constraints remain unchanged under all noise conditions.

To evaluate the robustness of the proposed model to observational noise, zero-mean Gaussian white noise with noise levels of 2% and 3% was added to the original gravity anomaly data, while the noise-free data were used as the reference case. Under different noise conditions, the well-logging constraints were kept unchanged so that the influence of noise in the gravity anomaly data on the inversion results could be examined independently. The reference density model used for testing is shown in Figure 10, and the gravity anomaly maps and corresponding inversion results under different noise levels are presented in Figure 11. As shown in Figure 11, under the noise-free condition, the model accurately recovers the principal spatial location and overall geometry of the anomalous body. The predicted anomaly is relatively compact, with a comparatively clear boundary. After 2% Gaussian white noise is added, the gravity anomaly contours exhibit a certain degree of perturbation. Nevertheless, the principal anomalous body can still be effectively identified, and its spatial location remains generally consistent with that obtained under the noise-free condition. However, the boundary of the anomalous body becomes less regular, and local structural deviations begin to appear.When the noise level increases to 3%, the local fluctuations in the gravity anomaly become more pronounced. Although the position and overall geometry of the principal anomalous body remain identifiable to some extent, relatively obvious discrete spurious anomalies and shallow anomalous bodies appear in the inversion result. In addition, the continuity and boundary integrity of the recovered anomalous body deteriorate. These results indicate that, as the observational noise level increases, the model predictions are progressively affected, resulting in a gradual decline in inversion accuracy.

Overall, the proposed model is capable of preserving the principal location and overall structure of the anomalous body under low-noise conditions, demonstrating a certain degree of noise robustness. However, under relatively high noise levels, the model tends to produce local structural deviations and spurious anomalies, indicating that it remains sensitive to stronger observational noise. In future work, the stability of the model under complex noise conditions could be further improved by introducing noise augmentation at different levels during training, increasing the diversity of noisy samples, or incorporating targeted regularization constraints.

### 3.2. Field Example

**Figure 12:**
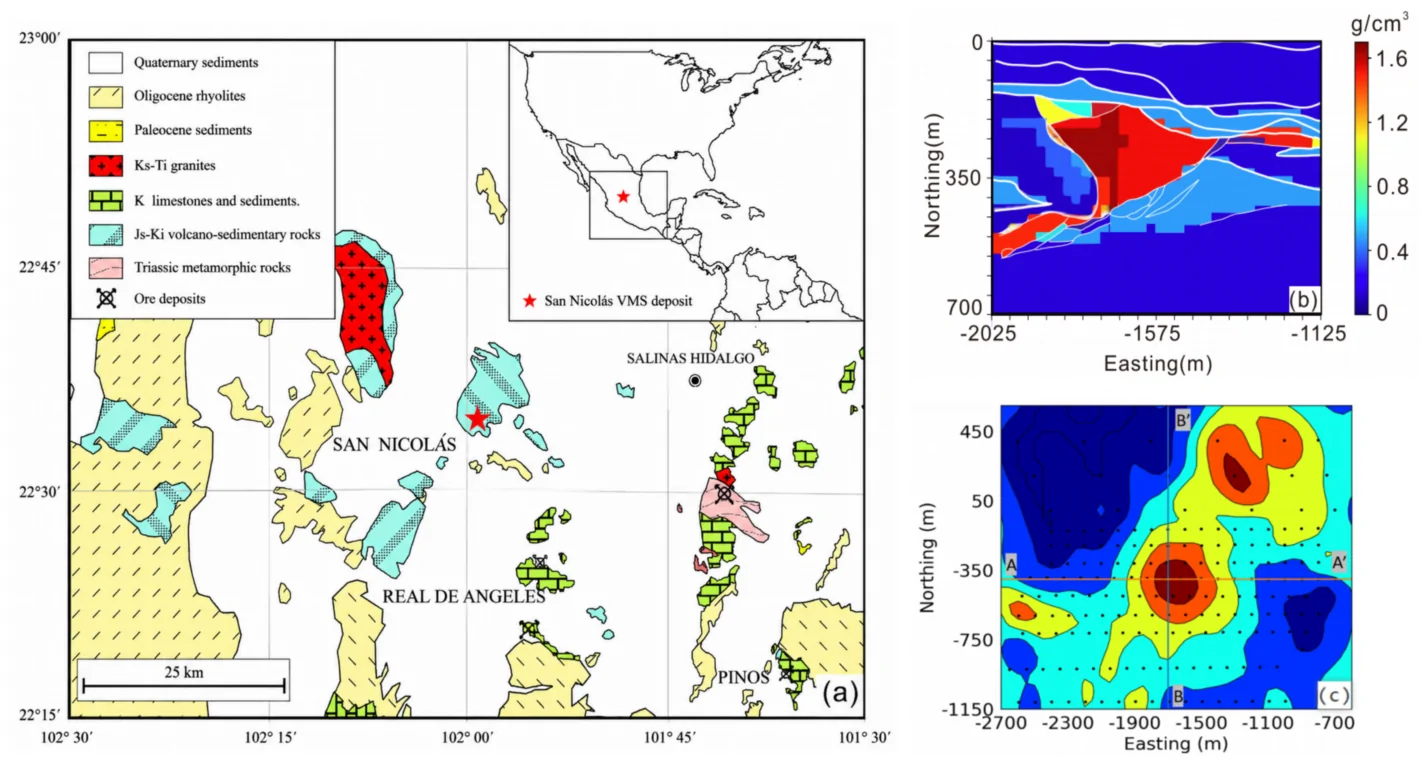
(**a**) Location and regional geological setting of the San Nicolás deposit. The red stars indicate the location of the San Nicolás VMS deposit, the inset shows its location in Mexico; (**b**) anomalous density model constructed from the geological model and physical-property information for the San Nicolás deposit, shown along a W–E cross-section at Northing =−400 m, with interfaces in the geological model highlighted in white; (**c**) Kriging-interpolated gridded residual gravity anomaly map. Panel (**a**) was adapted from Figure 2 of Ref. [36] under the Creative Commons Attribution 4.0 International License (CC BY 4.0); the original figure was cropped and rearranged. Panel (**b**) was adapted with permission from Figure 20a of Ref. [38]; the representation of the tick-mark labels was modified. Copyright 2009 The Authors; journal compilation copyright 2009 Royal Astronomical Society. Panel (**c**) was generated by the authors using the code and data associated with Ref. [19] and the accompanying Zenodo repository (DOI: 10.5281/zenodo.5211231), licensed under CC BY 4.0.

Figure 12a, adapted from Vassallo et al. [36], illustrates the location and regional geological setting of the San Nicolás deposit in Zacatecas State, Mexico. The San Nicolás deposit is a volcanogenic massive sulfide (VMS) deposit containing gold, silver, copper, and zinc [36,37]. The sulfide ore body has a density of approximately 3.5 g/cm^3^ and occurs within the contact zone between the upper rhyolitic dome and the underlying volcano-sedimentary sequence [38,39]. As shown in Figure 12b, the top of the massive sulfide ore body occurs at a depth of approximately 150–220 m, whereas its base extends to approximately 400–450 m [38]. The ore body produces the principal gravity anomaly near the center of the survey area, with a maximum amplitude of approximately 2.2 mGal, while the remaining anomalies are mainly associated with mafic volcanic rocks [38,39]. As summarized in Table 2, the sulfide has a density of approximately 3.5 g/cm^3^, whereas the densities of the principal host rocks range from approximately 2.3 to 2.7 g/cm^3^ [39]. Accordingly, a representative density contrast of approximately 1.1 g/cm^3^ was adopted for the study area.

To satisfy the input requirements of the UCA-W network for normalized matrix elements, we performed kriging interpolation on the irregularly distributed Bouguer gravity anomaly data shown in Figure 12c, generating a uniformly gridded gravity anomaly dataset. Subsequently, we conducted gravity inversion to reconstruct the subsurface density distribution.

**Figure 13:**
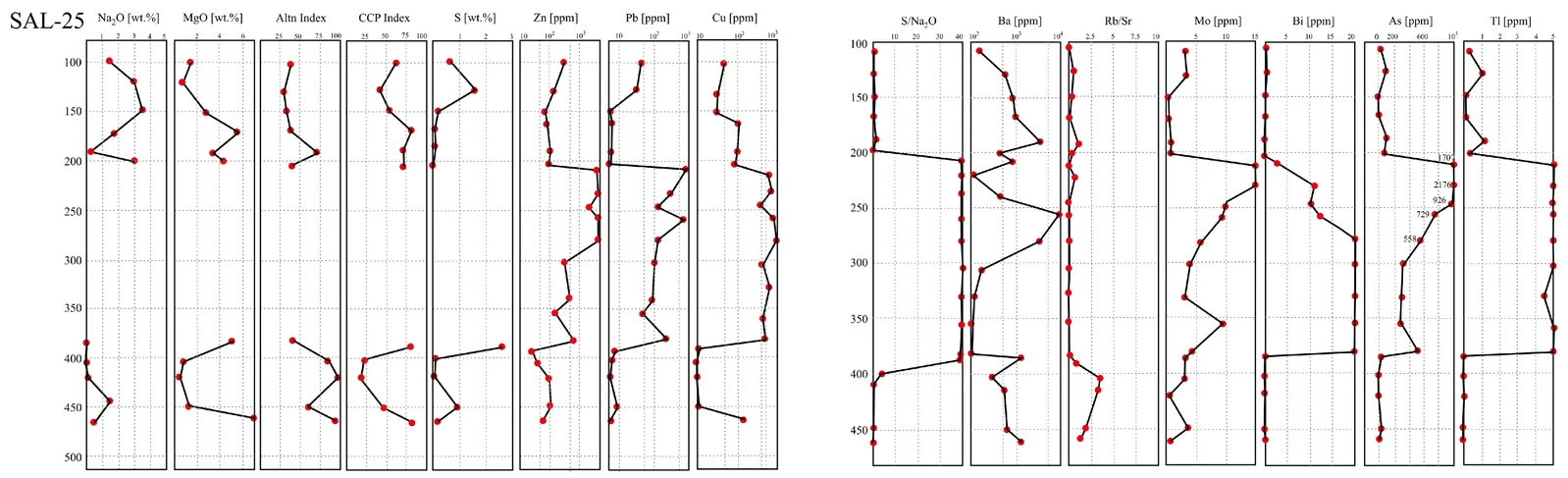
Geologic logs and geochemical data for the San Nicolás SAL-25 drill hole through the hanging-wall and footwall alteration zones. Reprinted without modification from Figure 14 of Ref. [36]. © 2015 Vassallo et al., licensed under the Creative Commons Attribution 4.0 International License (CC BY 4.0).

In the comprehensive investigation of the subsurface structure in the San Nicolas area, Vassallo et al. [36] provided crucial well-logging data that significantly enhanced our understanding of the regional geological characteristics. Figure 13 presents the geological data acquired from the SAL-25 drill hole.

**Figure 14:**
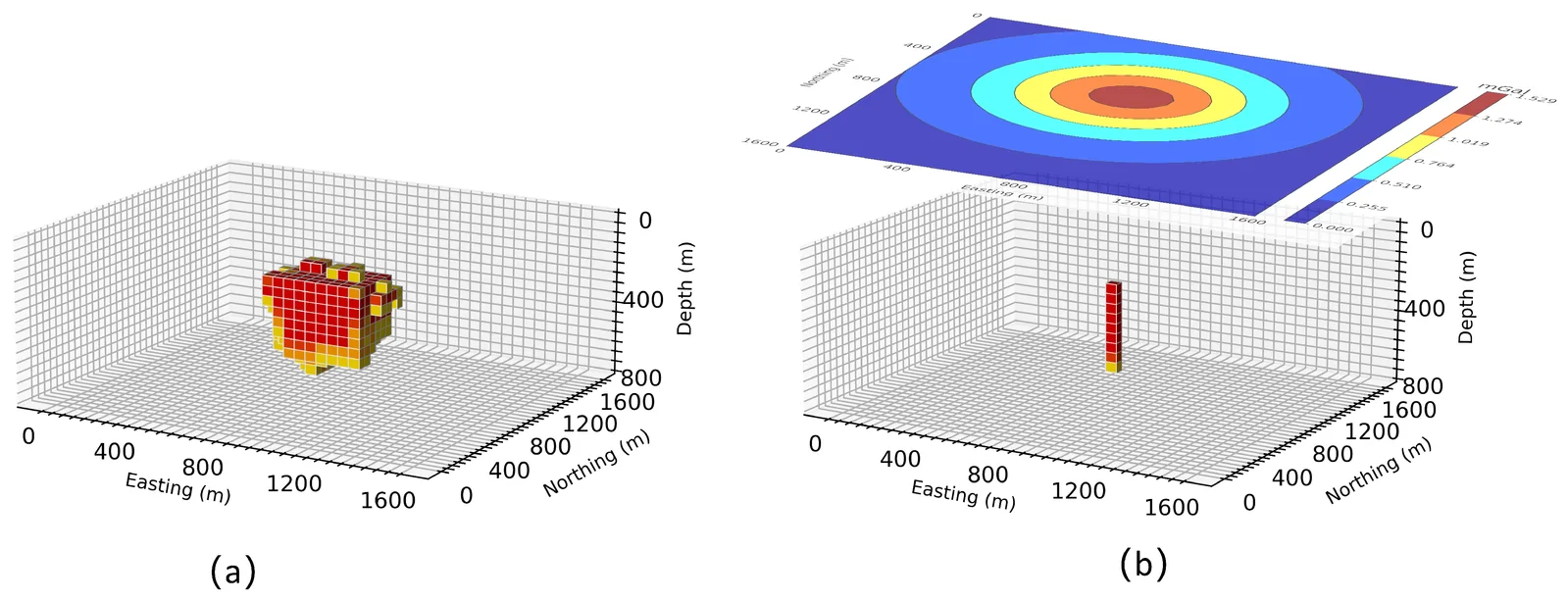
Representative samples from the San Nicolas mining area dataset. (**a**) Density distribution of a geological body; (**b**) corresponding gravity anomalies with well-logging constraints.

Prior to practical deposit prediction, although the datasets used in synthetic experiments demonstrated the superiority of the UCA-W network, these datasets might not fully represent the geological features of the target area under actual conditions. Therefore, a field-calibrated dataset was constructed to reduce the discrepancy between the general synthetic dataset and the petrophysical characteristics of the San Nicolas mining district. The anomalous-body geometries in this dataset were generated using the same stochastic random-walk procedure adopted for the general synthetic dataset, while the spatial discretization and sample-generation workflow remained unchanged. The field-specific adaptation was limited to the density parameters derived from the geological and petrophysical information of the study area. Specifically, the principal residual density of the anomalous bodies was set to 1.1 g/cm^3^, instead of the value of 1.0 g/cm^3^ used in the general synthetic dataset. In addition, residual-density variations ranging from 0.8 to 1.1 g/cm^3^ were introduced in the marginal zones of the anomalous bodies to represent gradual petrophysical variations near their boundaries, as illustrated in Figure 14.

It should be emphasized that the field-calibrated dataset did not incorporate the exact geometry, spatial position, or boundaries of the actual orebody as training labels. Its purpose was only to adapt the density distribution of the synthetic samples to the petrophysical characteristics of the study area. Moreover, both UCA-W and UNet-W were fine-tuned using the same field-calibrated dataset and identical training settings. Therefore, the dataset adaptation contributed equally to both networks, whereas the performance difference between UCA-W and UNet-W under the same data conditions primarily reflects the contribution of the proposed well-logging integration and attention-guided feature-fusion strategy.

**Figure 15:**
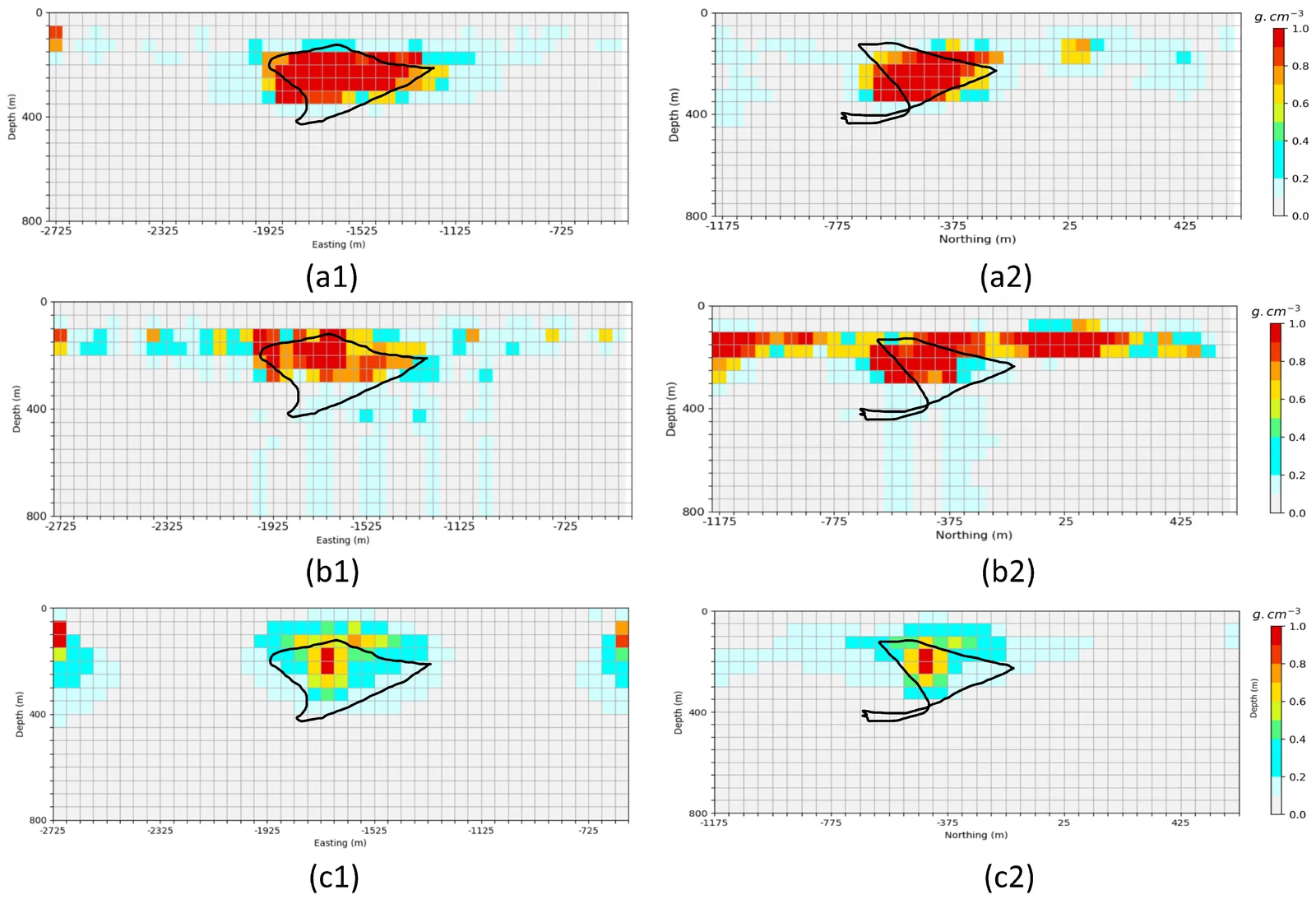
Vertical cross-sections of inversion results from the San Nicolas deposit. (**a1**) Northing = −400 m section via UCA-W; (**a2**) Easting = −1700 m section via UCA-W; (**b1**) Northing = −400 m section via UNet-W; (**b2**) Easting = −1700 m section via UNet-W; (**c1**) Northing = −400 m section via PCG; (**c2**) Easting = −1700 m section via PCG. Black contours denote actual orebody boundaries.

Following parameter fine-tuning using the new dataset, we inverted the sulfide orebody using the interpolated gravity anomaly data and the prior constraints from the SAL-25 well. Figure 15 presents the inversion results obtained from the UCA-W network, the UNet-W network, and the traditional preconditioned conjugate gradient (PCG) method incorporating well-log density information for the measured data from the deposit. The black contours represent the actual orebody boundaries. Panels (a1), (b1), and (c1) show the vertical cross-sections obtained using the UCA-W network, the UNet-W network, and the PCG method, respectively, at Northing =−400 m, whereas panels (a2), (b2), and (c2) show the corresponding vertical cross-sections at Easting =−1700 m. As shown in Figure 15, the prediction distribution produced by the proposed UCA-W network is highly concentrated, and the predicted location is generally consistent with the borehole information. Meanwhile, the predicted geometry and physical boundaries of the anomalous source agree well with the actual conditions. These results are consistent with those obtained from our previous synthetic experiments, confirming that the fine-tuned UCA-W network model can maintain relatively high prediction accuracy and stability when dealing with complex real-world geological scenarios. In contrast, although the traditional preconditioned conjugate gradient (PCG) method can approximately recover the geometric outline of the anomalous source, the recovered physical-property parameters, namely the density values, are generally underestimated.

## 4. Conclusions

In this study, we propose a novel UCA-W network architecture based on the UNet framework. The network simultaneously processes two-dimensional surface gravity anomalies and subsurface well-logging data, with the latter serving as hard constraints during the inversion process. Notably, we integrate the Convolutional Block Attention Module (CBAM) into the downsampling pathway, which enhances the model’s ability to capture critical spatial features and structural details of geological formations. This innovative design significantly improves the network’s performance while ensuring that the inversion results exhibit excellent agreement with the true subsurface configuration in terms of fine-scale characteristics.

The incorporation of well-logging constraints enables the deep learning model to achieve more accurate characterization of subsurface geological structures, thereby enhancing both the precision and reliability of the inversion results. The effectiveness of our approach has been comprehensively validated through synthetic modeling tests and field application to the San Nicolas mining area in Mexico. Future research should focus on developing more efficient strategies for utilizing well-logging information and designing more robust network architectures to further optimize inversion performance.

## Figures and Tables

**Table 1:** Comparative performance evaluation of different inversion methods.

Method	IOU	ACC	MAE	SSIM
UNet-W	0.67	0.85	0.0160	0.7650
SSGI-W	0.56	0.78	0.0236	0.6339
UCA-W	0.75	0.93	0.0094	0.8663
UCA	0.71	0.88	0.0100	0.8351

**Table 2:** Physical properties of major rock units in the San Nicolas Deposit.

Rock Type	Density (g/cm^3^)
Tertiary Breccia	2.3
Mafic Volcanic Rock	2.7
Sulfide	3.5
Quartz Rhyolite	2.4
Graphitic Shale	2.1

## Data Availability

The data that support the findings of this study are available from the corresponding author upon reasonable request.

## References

[1] Camacho A.G., Prieto J.F., Aparicio A., Ancochea E., Fernandez J. (2021). Upgraded Growth 3.0 Software for Structural Gravity Inversion and Application to El Hierro (Canary Islands). Comput. Geosci..

[2] Sjöberg L.E., Bagherbandi M. (2017). Gravity Inversion and Integration.

[3] Geng M., Huang D., Yang Q., Liu Y. (2014). 3-D Inversion of Airborne Gravity-Gradiometry Data Using Cokriging. Geophysics.

[4] Zhang J., Wang C.-Y., Shi Y., Cai Y., Chi W.-C., Dreger D., Cheng W.-B., Yuan Y., Yuan Y.-H. (2004). Three-Dimensional Crustal Structure in Central Taiwan from Gravity Inversion with a Parallel Genetic Algorithm. Geophysics.

[5] Liu S., Hu X., Liu T. (2014). A Stochastic Inversion Method for Potential Field Data: Ant Colony Optimization. Pure Appl. Geophys..

[6] Nagihara S., Hall S.A. (2001). Three-Dimensional Gravity Inversion Using Simulated Annealing: Constraints on the Diapiric Roots of Allochthonous Salt Structures. Geophysics.

[7] Pallero J.L.G., Fernández-Martínez J.L., Bonvalot S., Fudym O. (2015). Gravity Inversion and Uncertainty Assessment of Basement Relief via Particle Swarm Optimization. J. Appl. Geophys..

[8] Feng D., Su X., Wang X., Zhu L., Yang J., Liu J., Xu C. (2024). An Efficient Inversion Framework for Audio-Magnetotellurics with Borehole Constraints Combining Supervised Descent Method and Gaussian Distribution Modeling Strategy. IEEE Sens. J..

[9] Puzyrev V. (2019). Deep Learning Electromagnetic Inversion with Convolutional Neural Networks. Geophys. J. Int..

[10] Wang Y., Ge Q., Lu W., Yan X. (2020). Well-Logging Constrained Seismic Inversion Based on Closed-Loop Convolutional Neural Network. IEEE Trans. Geosci. Remote Sens..

[11] Wu Y., Lin Y. (2019). InversionNet: An Efficient and Accurate Data-Driven Full Waveform Inversion. IEEE Trans. Comput. Imaging.

[12] Yang F., Ma J. (2019). Deep-Learning Inversion: A Next-Generation Seismic Velocity Model Building Method. Geophysics.

[13] Huang R., Zhang Y., Vatankhah S., Liu S., Qi R. (2022). Inversion of Large-Scale Gravity Data with Application of VNet. Geophys. J. Int..

[14] Zhang Y., Li H., Qiu M. (2024). 3-D Gravity Anomaly Forward and Inversion Algorithm Based on Improved LinkNet. Prog. Geophys..

[15] Zhou S., Wei Y., Lu P., Jiao J., Jia H. (2024). Deep-Learning Gravity Inversion Method with Depth-Weighting Constraints and Its Application in Geothermal Exploration. Remote Sens..

[16] Yang Q., Hu X., Liu S., Jie Q., Wang H., Chen Q. (2021). 3-D Gravity Inversion Based on Deep Convolution Neural Networks. IEEE Geosci. Remote Sens. Lett..

[17] Zhang Z., Liao X., Cao Y., Hou Z., Fan X., Xu Z., Shi Z. (2021). Joint Gravity and Gravity Gradient Inversion Based on Deep Learning. Chin. J. Geophys..

[18] Zhang L., Zhang G., Liu Y., Fan Z. (2021). Deep Learning for 3-D Inversion of Gravity Data. IEEE Trans. Geosci. Remote Sens..

[19] Huang R., Liu S., Qi R., Zhang Y. (2021). Deep Learning 3-D Sparse Inversion of Gravity Data. J. Geophys. Res. Solid Earth.

[20] Lv M., Zhang Y., Liu S. (2023). Fast Forward Approximation and Multitask Inversion of Gravity Anomaly Based on UNet3+. Geophys. J. Int..

[21] Joulidehsar F., Moradzadeh A., Doulati Ardejani F. (2018). An Improved 3-D Joint Inversion Method of Potential Field Data Using Cross-Gradient Constraint and LSQR Method. Pure Appl. Geophys..

[22] Zhang R., Li T., Liu C., Huang X., Jensen K., Sommer M. (2021). 3-D Joint Inversion of Gravity and Magnetic Data Using Data-Space and Truncated Gauss–Newton Methods. IEEE Geosci. Remote Sens. Lett..

[23] Fang Y., Wang J., Meng X., Zheng S., Tang H. (2022). An Efficient Cross-Gradient Joint Inversion Algorithm for Gravity and Magnetic Data Using a Sequential Strategy. IEEE Trans. Geosci. Remote Sens..

[24] Chasseriau P., Chouteau M. (2003). 3-D Gravity Inversion Using a Model of Parameter Covariance. J. Appl. Geophys..

[25] Shamsipour P., Marcotte D., Chouteau M., Keating P. (2010). 3-D Stochastic Inversion of Gravity Data Using Cokriging and Cosimulation. Geophysics.

[26] Sun J., Li Y. Inversion of Surface and Borehole Gravity with Thresholding and Density Constraints. Proceedings of the SEG Technical Program Expanded Abstracts 2010.

[27] Qiao Z.-K., Zhang Z.-Y., Hu R., Shen Z.-H., Yuan P., Zhou H. (2024). Joint Inversion of Gravity and Gravity Gradient Data Based on Cross-Gradient Function. IEEE Sens. J..

[28] Li Y., Jia Z., Lu W. (2022). Self-Supervised Deep Learning for 3-D Gravity Inversion. IEEE Trans. Geosci. Remote Sens..

[29] Chen T., Zou B., Wang Y., Cai H., Li X., Yu G., Hu G. (2024). Prestack Seismic Inversion Driven by Priori Information Neural Network and Statistical Characteristic. IEEE Trans. Geosci. Remote Sens..

[30] Kim S., Cho Y., Gihm Y., Jun H. (2025). Machine Learning-Based Prediction of Subsurface Elastic Properties Using Seismic and Well-Log Data. IEEE J. Sel. Top. Appl. Earth Obs. Remote Sens..

[31] Song C., Lu W., Ye S., Yao W., Zhang X. (2026). Multimodal Geophysics-Informed Neural Network for Joint Inversion of Seismic, Electromagnetic, and Well-Logging Data. IEEE Trans. Geosci. Remote Sens..

[32] Zhdanov M.S. (2002). Geophysical Inverse Theory and Regularization Problems.

[33] Zhang L., Zhang G., Fan Z., Ma J. (2022). A Multitask Deep Learning for Simultaneous Denoising and Inversion of 3-D Gravity Data. IEEE Trans. Geosci. Remote Sens..

[34] Ronneberger O., Fischer P., Brox T. (2015). U-Net: Convolutional Networks for Biomedical Image Segmentation. Proceedings of the International Conference on Medical Image Computing and Computer-Assisted Intervention (MICCAI).

[35] Woo S., Park J., Lee J.Y., Kweon I.S. CBAM: Convolutional Block Attention Module. Proceedings of the European Conference on Computer Vision (ECCV).

[36] Vassallo L.F., Aranda-Gómez J.J., Solorio-Munguía J.G. (2015). Hydrothermal Alteration of Volcanic Rocks Hosting the Late Jurassic–Early Cretaceous San Nicolás VMS Deposit, Southern Zacatecas, Mexico. Rev. Mex. Cienc. Geológicas.

[37] Johnson B.J., Montante-Martínez J.A., Canela-Barboza M., Danielson T.J., Sherlock R.L., Logan M.A.V. (2000). Geology of the San Nicolás Deposit, Zacatecas, Mexico. VMS Deposits of Latin America.

[38] Lelièvre P.G., Oldenburg D.W. (2009). A Comprehensive Study of Including Structural Orientation Information in Geophysical Inversions. Geophys. J. Int..

[39] Phillips N., Oldenburg D., Chen J., Li Y., Routh P. (2001). Cost Effectiveness of Geophysical Inversions in Mineral Exploration: Applications at San Nicolas. Lead. Edge.

